# The aged worker in contemporaneity

**DOI:** 10.1590/1518-8345.0000.3471

**Published:** 2021-05-21

**Authors:** Aida Maria de Oliveira Cruz Mendes, Maria Lucia do Carmo Cruz Robazzi

**Affiliations:** 1 Escola Superior de Enfermagem de Coimbra, Unidade de Investigao em Cincias da Sade: Enfermagem, Coimbra, Portugal.; 2 Universidade de So Paulo, Escola de Enfermagem de Ribeiro Preto, PAHO/WHO Collaborating Centre for Nursing Research Development, Ribeiro Preto, SP, Brazil.; 3 Universidade Federal da Paraba, Joo Pessoa, PB, Brazil.

**Figure:**
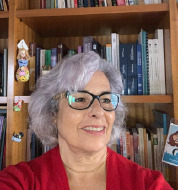

**Figure:**
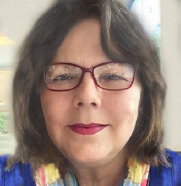

Workers health is a field of wide and complex knowledge that studies and intervenes both in the health-protective factors of those who work, aiming at health promotion, and in those that can influence their illness, in a preventive perspective and for the improvement of the working conditions.

In the scope of this heterogeneity, diversified types of work environments are found, presenting countless occupational risk factors, in addition to many categories of workers in activities, belonging to different genders and age groups. Among the age variations of the individuals present in the labor market are the older adults.

There is, then, a current trend to extend the working life, justified by economic reasons, both individual due to survival needs and global due to insufficiency of the assistance systems, in different countries.

In Brazil, an individual is considered aged at 60 (sixty) years old or over and this definition is the same advocated by the World Health Organization (WHO)^(^ 1 ^)^. Nowadays, in the country there are more than 28 million people in this situation, representing 13% of the total population, with the percentage tending to double in the next decades, according to the 2018 Population Projection^(^ 2 ^)^.

Also in Brazil, the Labor Secretariat of the Ministry of Economy made available data from the Annual Report of Social Information (Relao Anual de Informaes Sociais, RAIS), showing that the number of people aged 65 years old or older with a formal contract increased from 484,000 in 2013, to 649,400 in 2017, showing a 43% expansion in four years^(^ 3 ^)^. In turn, in Portugal, in 2019, 17.3% of the active workers were between 55 and 64 years old and 5.3% were over 65; for the age group of 65 years old and older, the annual variation rate has been positive since 2014, with a more significant percentage increase in 2017, when it reached a 4.3% increase in relation to the previous year^(^ 4 ^)^.

This is a visible trend in many other countries. In Austria, for example, the proportions of working individuals aged 55-59 and 60-64 are 67% and 27%, respectively; in Canada, 71% and 51%; in Denmark, 81% and 55%; in France, 71% and 28%; in Germany, 79% and 56%; in the United States, 69% and 54%; and in Japan, 80% and 64%. In fact, the actual workforce participation rates of people aged 60 to 64 have been increasing in the G7 economies (Canada, Japan, United Kingdom, Italy, United States, Germany, and France) since the 2000s^(^ 5 ^)^.

These aged workers frequently have extensive work experience, maturity, and skill; and their acceptance at work seems to increase, since the population, in general, is also aging^(^ 3 ^)^. But, on the other hand, discrimination reactions persist in relation to them, because some tasks become difficult for them to perform, especially if there is a need for physical strength. In the labor market, they need to compete with the young and qualified individuals and face social stigmatization, which hinders their work permanence, in addition to being overlooked in job positions in relation to their younger competitors; in some companies, employees are fired when they are about to complete the necessary time for retirement; and bad jokes and moral harassment against these workers are a reality, among other facts^(^ 6 ^)^.

Currently, the spread of SARS-CoV-2 imposes special attention to the health of these individuals. On one hand, because they constitute a group at a higher risk for the development of more severe forms of COVID-19 and, on the other hand, because they often find themselves unprotected in their ability to use many protection means, such as, for example, remote work. Nevertheless, some studies have shown that older workers are more effective in responding to the COVID-19 pandemic. Such apparently contradictory results remind of the role of age in a lesser reaction to stress, more resilience, and greater competence for emotional regulation, with greater use of preventive behaviors and strategies centered on the emotions^(^ 7 ^)^.

However, inter-individual differences increase with age, which is why different profiles of self-regulation strategies among the older workers must be considered^(^ 8 ^)^, which requires organizations and those who deal with Workers Health to have protection plans especially aimed at this age group.

The insertion of the older adult in the labor market is a contemporary fact and a right of the individual and, thus, age as a vulnerability factor and its uniqueness must be considered when studying and planning interventions related to Workers Health.
